# The impact of cancer campaigns in Brazil: a Google Trends analysis

**DOI:** 10.3332/ecancer.2019.963

**Published:** 2019-09-24

**Authors:** Luiz Fernando Quintanilha, Laumar Neves Souza, Daniel Sanches, Rafael Senos Demarco, Kiyoshi Ferreira Fukutani

**Affiliations:** 1 Universidade Salvador, Laureate Universities, Salvador 41770-235, Brazil; 2 Centro Universitário FTC, Faculdade de Medicina, Salvador 41741-590, Brazil; 3 Division of Arts and Sciences, South Florida State College, Avon Park, FL 33825, USA; 4 University of California at Los Angeles, Los Angeles, CA 90095, USA; 5 Instituto Gonçalo Moniz, Fundação Oswaldo Cruz, Salvador 40296-710, Brazil; 6 Multinational Organization Network Sponsoring Translational and Epidemiological Research (MONSTER) Initiative, Fundação José Silveira, Salvador 40210-320, Brazil

**Keywords:** neoplasms, health promotion, national health programmes, breast neoplasms, prostatic neoplasms

## Abstract

It is estimated that more than 600,000 new cases of cancer will be reported in Brazil during the 2018–2019 biennium, especially with regard to prostate, breast, lung and colorectal cancers. Due to the high prevalence, incidence and mortality rates of these diseases, cancer campaigns such as ‘Pink October’ and ‘Blue November’ were strongly promoted in the past decade throughout the country to raise awareness of breast and prostate cancer, respectively. Nevertheless, whether the implementation of these campaigns has been proven efficient is still unknown. In the present study, we analysed the effectiveness of these campaigns on eliciting population online interest for cancer information. The Google Trends database was evaluated for the relative Internet search popularity for the terms ‘breast cancer’ and ‘prostate cancer’ from 2014 to 2019. Aside from some regional differences, we found that there was a high demand for ‘breast cancer’ and, to a lesser extent, ‘prostate cancer’ searches in a seasonal fashion (during October and November, respectively). Despite the worldwide high incidence of lung and colorectal cancers, searches including these keywords did not show increases in any specific period of the year, demonstrating the efficiency of the ‘Pink October’ and ‘Blue November’ campaigns in engaging the interest of the Brazilian population on the subject. These results allow us to infer that campaigns are effective in mobilising the attention of the Brazilian population with regard to breast and prostate cancers, but the practical aspects in reducing incidence and mortality should still be discussed.

## Background

One of the most important phenomena that the world population has been experiencing over the past decades is the elevation of life expectancy. In fact, a recent World Health Organization (WHO) study showed that global life expectancy increased by 5.5 years (achieving 72.0 years) from 2000 to 2016. However, this progression did not occur homogeneously among all countries, with greater rates of life expectancy increase happening in developing countries [1]. In Brazil, particularly, there was a growth of 7 years, going from 68.8 to 75.8 years.

Increasing life expectancy is obviously a positive aspect, but some negative side effects may arise, such as higher incidence and development of age-related diseases such as cancer [2–4]. In this context, several studies have shown that molecular, cellular and physiological alterations associated with ageing are involved in carcinogenesis, contributing to the increase in prevalence, incidence and mortality [5, 6].

In recent years, an increase in the number of cases of this disease has been reported in all regions [7]. Based on the statistics provided by the WHO, it is estimated that in 2018, this disease claimed the lives of about 9.6 million people on the planet, which is 20% higher than in 2010 [8]. The severity and magnitude of this number can be understood, as it corresponds to 17% of all deaths (in other words, one in six deaths were related to neoplasia). By 2040, there will be 29.5 million people diagnosed with cancer worldwide (63.8% more than the 18 million people diagnosed today) that could have a significant social, economic and health impact [9].

According to the Brazilian National Cancer Institute (INCa), it is estimated that Brazil will have around 600,000 new cases of cancer in the 2018–2019 biennium. Among those, the most prevalent types are prostate (68,220 cases), breast (59,700 cases), colorectal (36,360 cases) and lung cancer (31,270 cases) [10]. In addition, there is a significant and growing number of hospitalisations and deaths related to those types of cancer [11].

In this scenario, campaigns were created in order to reduce the impacts of different types of cancer in the population. Examples of highly publicised campaigns in Brazil include ‘Pink October’, whose goal is to share information about breast cancer (and, more recently, cervical cancer as well), promoting disease awareness, providing greater access to diagnostic services and contributing to reduced mortality. Another example is the ‘Blue November’ campaign, which acts in a similar manner to ‘Pink October’, but focuses on prostate cancer. With the increase in institutions linked to the cause, and the growing exposure and popularity of the campaigns, it is expected that there will be more awareness among the population and an increase in the number of examinations for breast and prostate cancer.

Some studies discuss the implementation and effectiveness of these programmes, especially with regard to the effectiveness in reducing incidence and mortality, and generating demand that is not the focus of the exams [12, 13]. On the other hand, awareness campaigns regarding the incidence of the disease (as well as its main characteristics, clinical signs, diagnosis and treatment) can be useful for a large portion of the population. Thus, the analysis of populational interest in relation to the ‘Pink October’ and ‘Blue November’ campaigns can generate useful indicators of their effectiveness, and guide their development, expansion and efficiency for the near future.

Due to the large size of samples analysed in populational studies, traditional enquiry methods are expensive, time-consuming and often extremely complex. A viable, quick and inexpensive alternative to assessing populational interest in cancer-related campaigns is the quantification of Internet searches, which can be easily verified using the tool ‘Google Trends’. This tool, launched by Google in 2012, is a free access portal that analyses billions of searches daily on its pages, providing information delimited by search terms, location and time. Due to its potential, it has been used for epidemiological evaluation worldwide with reasonable success [14–18]. ‘Google Trends’ has also been used to correlate the amount of online searches on cancer with incidence and mortality rates of such disease [19–22].

In this sense, the goal of this study is to evaluate the impact of cancer-related campaigns on the interest of the Brazilian population by analysing online relative search volume (RSV) performed in the past 5 years.

## Methods

Google Trends (https://trends.google.com/trends/?geo=US), a free web-based tracking system, was used to determine Google search volumes. The data are presented as RSV, which is computed as the percentage of searches of a term in a location during a specific period. This RSV value means the ratio between the specific topic and the total amount of Google queries.

The Google Trends tool normalises data by using the highest query share of that term over the historical series. Each data point is divided by the total searches performed in the geographical region and time range it represents in order to compare the relative popularity of such point. Values are displayed on a scale of 0–100, where 100 is the most popular place as a fraction of the total searches in that place, and 0 indicates a place where there was not enough data for the term. A larger value means a higher proportion of queries (not a higher absolute count) that allow us to compare different regions regardless of the absolute number of online searches. In this sense, higher RSV values indicate where the term is most likely to be searched.

For this study, the Google Trends tool was used to explore Internet activity related to breast and prostate cancers. For comparison, the terms lung and colorectal cancers were also evaluated due to the high prevalence rates of these diseases, but a lack of specific widespread awareness campaigns targeting them. The selected categories were ‘health’ and ‘web searches’, and the country of ‘Brazil’ was selected as the region of interest. This study focussed on evaluating public interest in Brazil in a 5-year period (from May 2014 to May 2019). The incidence (or ‘popularity’) of the search terms was compared among the five geographical regions that constitute Brazil: North, Northeast, Midwest, Southeast and South; additional comparisons were done within all states that comprise different Brazilian regions. We used the Portuguese terms for ‘breast cancer’ (‘cancer de mama’), ‘prostate cancer’ (‘cancer de prostata’), ‘lung cancer’ (‘cancer de pulmao’) and ‘colorectal cancer’ (‘cancer colorretal’).

The temporal graph was made using the values of RSV in each month depicted in a line graph built with ggplot2 package [23]. The Brazilian heatmap was built with the tmap package [24].

The present study followed standard ethical norms. No further approval requests to the Research Ethics Committee were needed, as only secondary and public domain data were used.

## Results

### Interest trends in breast, prostate, lung and colorectal cancers among Brazilians

**Figure 1.**
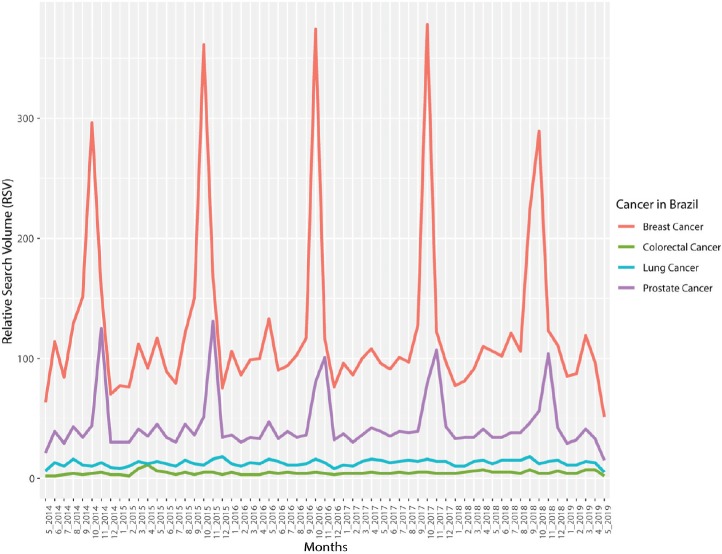
Breast, prostate, lung and colorectal cancer RSV from May 2014 to May 2019 [Source: Google Trends, 2019].

The analysis of the Brazilian online interest on these terms by Google Trends showed a predominance for the term ‘breast cancer’, followed by the terms ‘prostate cancer’, over ‘lung cancer’ and ‘colorectal cancer’. Interestingly, although ‘breast cancer’ is not the most prevalent type of cancer in the Brazilian population [10], it was the type of cancer with the most interest among Brazilians, with values about three times higher than the demand for prostate cancer information (Figure 1).

### Differences in online-seeking pattern among Brazilian regions

**Figure 2.**
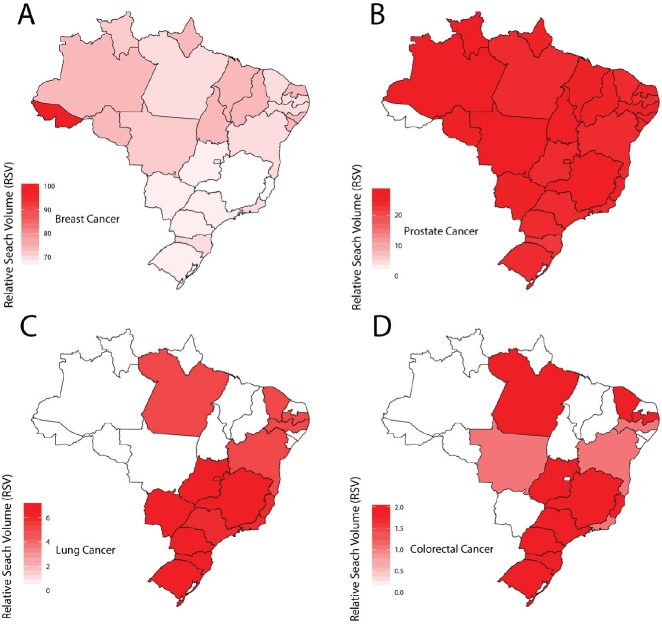
Brazilian heat map showing the distribution of population interest by Google Trends RSV information for (A): breast, (B): prostate, (C): lung and (D): colorectal cancer among regions of Brazil, May 2014–May 2019 [Source: Google Trends, 2019].

When evaluating the Brazilian regions separately, RSV values for breast cancer were the highest in all regions, followed by prostate, lung and colorectal cancers. It is interesting to note that breast and prostate cancer terms presented high RSV values in the North (76.4% for ‘breast cancer’ and 22.6% for ‘prostate cancer’ cases, respectively) and the Northeast (72% for breast and 20.5% for prostate, respectively). Significantly less focus was observed for lung (North: 0.7%, Northeast: 1.8%) and colorectal cancer (North: 0.3%, Northeast: 0.5%) in these regions (Figure 2).

These numbers are quite different from the results acquired from the South and Southeast regions of Brazil (both of which concentrate most of the country’s wealth). In these regions, there is still a predominance of breast and prostate cancers, but colorectal and lung cancers apparently have more prominence when compared to the North and Northeast regions. For example, when comparing the RSV values between the North and South regions, the relative value for colorectal cancer was about seven times higher than that registered for the North region. An even more discrepant picture is observed when information about lung cancer search is compared, since the RSV for the South region is ten times greater than that for the North. These results reveal a discrepancy among Brazilian regions with regard to the popularity of these search terms (Figure 2). Discrepancies are also observed among states, such as Acre (in the North), where breast cancer presents an RSV of 100, while Minas Gerais (in Southeast) has an RSV of 64 (Table 1).

## Discussion

As expected, a seasonality in the search for ‘prostate cancer’ and ‘breast cancer’ terms in the last 5 years was clearly noticeable (Figure 1). ‘Prostate cancer’ screening is concentrated in November, while ‘breast cancer’ is concentrated in October. Similar results were recently reported by Mohamad and Kok [25] in Malaysia. Such behaviour of the population on the Internet is most likely linked to the existence of the ‘Pink October’ and ‘Blue November’ campaigns. Thus, it seems that the campaigns have a strong effect on the interest of the population by the theme, reflected by the increase in Internet volume search. In this sense, media coverage seems to be an important explanatory factor [26]. An example of how the media can affect this analysis was recently presented by Faoury *et al* [27] and colleagues this year, in which throat cancer searches presented three peaks during the analysed period and these peaks were mostly related to a famous actor’s throat cancer diagnosis and news related to this case.

We cannot, however, ignore other aspects related to the analysis by Google Trends. It was shown that economic and health statuses, for example, have influence on Internet usage and health-related search patterns among regions and countries [28]. Another recent study showed that online interest in prostate cancer varies through time, and it can be related to scientific discoveries and changes in medical guidelines [29]. In addition, a study in the United States has shown that general interest in skin cancer information increases during the summer months, revealing that factors such as natural conditions, incidence of the disease, media coverage (particularly when celebrity personalities are involved) and informational campaigns can greatly affect population interest on certain themes [21, 30]. Together, these data highlight how media coverage is an important contributing factor for the rise in awareness and interest among the population, though other variables must be considered when Google Trends is used for epidemiological purposes.

It is interesting to note, however, the high rate of searches for the term ‘breast cancer’ in Brazil. Even though lung cancer is the most prevalent type of cancer worldwide [8], and prostate cancer has a higher prevalence and mortality rate than breast cancer in Brazil [10], ‘breast cancer’ searches were almost three-fold higher than any other online search category here analysed (Figure 1). This fact could potentially be explained by the intense media coverage on the topic during the ‘Pink October’ campaign, to the detriment of the ‘Blue November’ campaign (which starts after the former), yielding less dissemination and awareness to the latter due to ‘attention saturation’ of the population. In addition, the fact that women are more prone to preventative care measures than men (such as periodic consultations with a gynaecologist from a young age [31]) likely contributes to the differences observed in Internet search interests (Figure 1). Also notably, the data displayed in Table 1 show that cancer awareness and information campaigns affect the regions of the country differently. This could potentially be explained by the unequal exposure of the campaigns in these regions, as well as differences in socioeconomic and educational levels, or even Internet access [32].

An increase in population-level interest regarding breast cancer has both positive and negative consequences. While an increase in information access leads to greater awareness in the population regarding certain types of diseases and how to treat/prevent them, it also takes a toll on the public health system, with nontarget groups (i.e., nonrisk groups) at times overloading the public health system. For instance, while the ‘Pink October’ campaign may have been successful in increasing early diagnosis while reducing mortality rates associated with breast cancer, it also results in increased demand—and consequentially costs—related to mammography exams (due to the fact that nontarget women are also worried about the incidence of breast cancer).

Notably, despite its advantages, the Google Trends tool presents some important limitations in the topic studied. Perhaps one of the most critical limitations is the fact that online searches are carried out mainly by a young public, in opposition to the onset of people affected by cancer (mostly middle aged people [2, 33, 34]). In addition, this tool uses data normalised by the number of Internet searches, hence considering only the portion of the population that has Internet access. This limitation may explain, for instance, the widely different RSVs found among different geographical regions of Brazil. Nevertheless, despite its limitations, the use of Google Trends to determine the effectiveness of health-related campaigns in Brazil (and potentially worldwide) can contribute to the improvement of methodologies and strategies utilised by these campaigns.

## Conclusions

Our results suggest that the ‘Blue November’ and (especially) ‘Pink October’ campaigns can mobilise the population to search for information regarding prostate and breast cancers, respectively, on the Internet. Based on Internet searches, our analyses may serve as an indicator of efficiency for health-related campaigns in Brazil. For instance, the data analysed here imply that there is a need for improving strategies during the ‘Blue November’ campaign in order to reach a greater audience (with respect to Internet searches). Moreover, this study also points to the current lack of interest among Internet users in lesser advertised cancer types, such as lung and colorectal cancers, raising the possibility for the development of newer and more effective campaigns targeting these highly prevalent diseases.

## Conflicts of interest

The authors declare that they have no conflicts of interest.

## Funding statement

This study has no public or private funding.

## Authors’ contributions

LF Quintanilha and KF Fukutani conceived of the presented idea. LF Quintanilha and KF Fukutani developed the theory and performed the computations. KF Fukutani, LN Souza and KF Fukutani verified the analytical methods. D Sanches, RS Demarco and LN Souza contributed to the final version of the manuscript. All authors discussed the results and contributed to the final manuscript.

## Figures and Tables

**Table 1.** Descriptive data of RSV by Google Trends according to selected cancers, states and regions of Brazil, May 2014–May 2019.

	Breast Cancer	Prostate Cancer	Lung Cancer	Colorectal Cancer
Acre	100	0	0	0
Amapá	74	26	0	0
Amazonas	73	27	0	0
Pará	69	24	5	2
Rondônia	74	26	0	0
Roraima	72	28	0	0
Tocantins	73	27	0	0
North Region (N)	76.4	22.6	0.7	0.3
Alagoas	75	25	0	0
Bahia	70	24	5	1
Ceará	69	24	5	2
Maranhão	74	26	0	0
Paraíba	69	24	5	2
Pernambuco	69	25	5	1
Piauí	74	26	0	0
Rio Grande do Norte	74	26	0	0
Sergipe	74	26	0	0
Northeast Region	72.0	25.1	2.2	0.7
Distrito Federal	69	25	6	0
Goiás	67	24	7	2
Mato Grosso	72	27	0	1
Mato Grosso do Sul	68	25	7	0
Midwest Region	69.0	25.3	5.0	0.8
Espírito Santo	66	26	6	2
Minas Gerais	64	27	7	2
Rio de janeiro	69	24	6	1
São Paulo	68	24	6	2
Southeast region	66.8	25.3	6.3	1.8
Paraná	68	23	7	2
Rio Grande do Sul	68	23	7	2
Santa Catarina	69	22	7	2
South Region	68.3	22.7	7.0	2.0

Source: Google Trends, 2019.
